# mHealth in Sub-Saharan Africa

**DOI:** 10.1155/2013/482324

**Published:** 2013-12-04

**Authors:** Thomas J. Betjeman, Samara E. Soghoian, Mark P. Foran

**Affiliations:** ^1^Ben Gurion University of the Negev, Medical School for International Health, New York, NY 10032, USA; ^2^NYU School of Medicine, Bellevue Hospital Center, New York, NY 10016, USA

## Abstract

Mobile phone penetration rates have reached 63% in sub-Saharan Africa (SSA) and are projected to pass 70% by 2013. In SSA, millions of people who never used traditional landlines now use mobile phones on a regular basis. Mobile health, or mHealth, is the utilization of short messaging service (SMS), wireless data transmission, voice calling, and smartphone applications to transmit health-related information or direct care. This systematic review analyzes and summarizes key articles from the current body of peer-reviewed literature on PubMed on the topic of mHealth in SSA. Studies included in the review demonstrate that mHealth can improve and reduce the cost of patient monitoring, medication adherence, and healthcare worker communication, especially in rural areas. mHealth has also shown initial promise in emergency and disaster response, helping standardize, store, analyze, and share patient information. Challenges for mHealth implementation in SSA include operating costs, knowledge, infrastructure, and policy among many others. Further studies of the effectiveness of mHealth interventions are being hindered by similar factors as well as a lack of standardization in study design. Overall, the current evidence is not strong enough to warrant large-scale implementation of existing mHealth interventions in SSA, but rapid progress of both infrastructure and mHealth-related research in the region could justify scale-up of the most promising programs in the near future.

## 1. Introduction

Mobile phones are increasingly accessible worldwide. There are an estimated 6.8 billion mobile phones being used in the world in 2013, compared to 1 billion in 2002, corresponding to penetration rates of approximately 96% globally: 128% in developed countries and 89% in developing countries [1]. In sub-Saharan Africa (SSA), the penetration of cell phones is estimated to be 63% in 2013 and projected to pass 70% by 2015 [2]. Hundreds of millions of people in SSA who never gained access to traditional landlines for telecommunication now use mobile phones on a regular basis [3]. In many developing countries, wireless technology is less expensive and more readily available than wired technology [4]. This technology has unique potential to reach large numbers of people living in resource-limited or remote locations.

Mobile health (mHealth) is the use of mobile phone technology for health-related purposes. This relatively new, dynamic, and rapidly evolving field includes the development and study of mobile phone applications such as short messaging service (SMS), voice calling, and wireless data transmission to collect or disseminate health-related information or to direct care. Recently, there has been an explosion in mHealth activities globally [5]. Early evidence from grey literature and peer-reviewed publications suggests that mobile phone-based platforms can be effectively and efficiently used for a variety of health-related purposes. However, the vast majority of published reports are program or project process evaluations. Scholarly articles describing health outcomes or analysing the cost-effectiveness of mHealth interventions remain few in number [6]. Further, there is a lack of standardization with regard to mobile device application design, which renders comparative studies looking at specific variables difficult [7]. Most mHealth research to date has been conducted in high-income countries with advanced mobile and information infrastructure. However, an increasing number of mHealth interventions are being developed and applied to disease prevention and control in more resource-limited contexts. Applications of mHealth within the field of global health include medication adherence, health worker communication, health education, and emergency and disaster response. Given its relatively large rural populations, varied landscape, limited transportation infrastructure, and rapidly expanding wireless network coverage, sub-Saharan Africa (SSA) is well positioned to benefit from the promise of remote medical service delivery. This review aims to provide a summary of key articles, published on or before March 16, 2013, from the peer-reviewed scientific literature describing current progress on mHealth initiatives in SSA and to explore potential future applications of mHealth in the region.

## 2. Methods

An initial search of original research and review articles was conducted via PubMed on March 16, 2013 using “mHealth” in the title or abstract. Since Professor Robert Istepanian first coined the term “mHealth” in 2004 [8], it has become a widely recognized and utilized term. The search was limited to this term to narrow the focus of the review. The initial search resulted in 109 articles. The articles were handsearched for reference to Sub-Saharan Africa, developing countries, or disaster response. This selection process yielded 21 articles. Articles not directly pertaining to health were removed, as were articles greater than ten-year old. This screening yielded 18 articles. Additional web searches provided background information relevant to the review. Articles were sorted by topic and findings summarized below.

## 3. Medication Adherence

Although many mHealth initiatives in SSA are still in the development and pilot phases, a growing number of applications have been implemented. To date, the majority of studies on mHealth interventions in the region have been small-scale pilot or feasibility studies evaluating SMS based messaging systems for improved disease management. Preliminary results are promising, especially in the area of medication adherence among patients being treated for HIV and/or tuberculosis (TB).

SIMpill is a pill dispensing system that embeds a SIM card in a small pill bottle, which registers and sends an SMS text to a central server each time the bottle is opened [9]. Each text message is time-stamped and contains a unique identification code linked to the patient's mobile phone number. If the central server does not receive text messages before a preset time, a reminder text is automatically sent to the patient's mobile phone. If there is no response, an alert is then sent to the patient's healthcare provider who can followup directly with the patient. A pilot study of 155 patients in Cape Town, South Africa, demonstrated treatment success rates of 94% with that system, up from 22–60%. SIMmed is a less expensive system that simply asks patients to dial into a central server each time they take their medications [9]. Again, if no communication is received from the patient by a designated time each day, the system sends a reminder text message to the patient, and alerts a healthcare provider if the patient again fails to respond. A SIMmed pilot showed compliance rates of 90%, but health outcomes such as treatment success rates have not yet been studied [9]. Nevertheless, such demonstrated improvements in treatment success rates with SIMpill and compliance rates with SIMmed are promising and could warrant further investigation and consideration on behalf of policy makers.

A similar product, Wisepill, was tested for technical feasibility and functionality in rural southwest Uganda [10]. Wisepill devices are also designed to promote medication adherence by transmitting data to a central server each time a patient's pill bottle is opened. The study compared use of a standard cellular network-based general packet radio service (GPRS) with one that added SMS service for real-time communication. The primary outcome tested was the number of network failures per person-month: (Network failures were defined as transmission interruptions of >48 hours due to lack of network connectivity). Among the 157 participants, there were 1.5 and 0.3 network failures per person-month for GPRS and GPRS + SMS, respectively. The additional cost for having SMS was approximately 1 USD per participant per month if one SMS was sent daily and GPRS was about 1.25 USD for the SIM card and 0.80 USD for 3 months of airtime to transmit data. The study concluded that the addition of SMS to cell networks serving remote areas significantly enhances the functionality of real-time mHealth applications in those areas [10]. Effects on patient adherence and treatment outcomes were not studied.

A mobile directly observed therapy (MDOT) model for tuberculosis studied in Kenya uses a combination of video and text messaging to encourage medication adherence [11]. The goal of this system is to improve treatment of TB among patients residing in rural locations and to decrease the burden of directly observed therapy (DOT) on patients and the healthcare system, without sacrificing effectiveness. The MDOT model has patient's relatives or friends capture video images of the patient taking his or her medication. Each day the new video is sent via mobile messaging service (MMS) to a central database where it is time and date stamped and logged. Medical nurses review the videos and can follow up if they were inadequate or not received. Video and SMS text messages containing health promotion messages and reminders are also sent out to patients regularly. A feasibility study in 13 patients found high degrees of interest and receptivity among both patients and nurses. However, about 50% of messages were not received, mostly as a result of technical issues and lost phones, and one patient was lost to follow-up [11]. In addition to these technical challenges, the high costs associated with MMS messaging and mobile phones capable of video communication may be a major limitation for the widespread adoption of this sort of system. However, current projections estimate that multimedia-messaging costs will decline and infrastructure will improve rapidly in the coming years. Further study with larger sample sizes and measurement of adherence and health outcomes may be warranted.

The WelTel Kenya1 study was designed to promote antiretroviral (ARV) medication adherence using a simpler and much less resource intensive system of weekly SMS text messages inquiring about patients' general wellbeing [12]. Patients are expected to respond within 48 hours. If a patient reports symptoms of poor health or does not respond, then healthcare providers follow up by phone. A randomised control trial of 538 participants, SMS intervention (*n* = 273) and standard care (*n* = 265), found significantly higher rates of self-reported medication adherence (relative risk for non-adherence 0.81; *P* < 0.006) and better rates of viral suppression (relative risk of viral load suppression failure 0.85; *P* < 0.04) in an intention to treat analysis [12]. The system costs under $8 USD per patient per year. Considering the cost of adding second-line therapy for patients who fail treatment, this application holds promise as a cost-effective strategy. Importantly, the WelTel Kenya1 trial did not incorporate specific daily or timed medication reminders and feedback, suggesting that improved communication and linking with healthcare providers alone may encourage patients to stick with long-term therapies.

Pop-Eleches et al. showed that automated text message reminders could be useful in improving HIV medication adherence in Kenya [13]. The study involved 431 adult patients who had initiated ARV treatment within 3 months. Participants in the intervention groups received SMS reminders that were either short or long and sent at a daily or weekly frequency. Adherence was measured using the medication event monitoring system on the pill bottle of one of the drugs prescribed and it was extrapolated that adherence with one drug most likely indicated adherence with all drugs. In intention-to-treat analysis, 53% of participants receiving weekly SMS reminders achieved adherence of at least 90% during the 48 weeks of the study, compared with 40% of participants in the control group (*P* < 0.03). Participants in groups receiving weekly reminders were also significantly less likely to experience treatment interruptions exceeding 48 hours during the 48-week follow-up period than participants in the control group (81 versus 90%, *P* < 0.03). Overall weekly short reminders seemed to be the most effective based on adherence rates in that subgroup. The results indicate that a simple and cheap intervention such as automated reminders for HIV patients on ARV therapy can significantly increase adherence. Further, this study suggests that short reminders are more effective than long reminders, which could inform future program design and effectiveness trails.

Based on the evidence from these short-term trails, SMS reminders could be a cost-effective way to improve medication adherence in SSA. This would especially be the case in areas that have preexisting wireless network coverage with SMS capabilities. Although much of the current research evaluating the impact of mHealth interventions on medication compliance in SSA has been promising, more work is needed to assess long-term impact. Longitudinal and follow-up studies will be an important addition to the current literature in this area.

## 4. Health Worker Communication

In addition to direct communication and support for patients, a number of mHealth applications have been designed as tools to increase community health worker (CHW) access to health information, decision making, and/or logistical support. For example, SMS-based communication and professional networking to support CHWs were studied in rural areas of Malawi [14]. The authors used a mixed methods approach to determine the frequency of SMS use, the most common reasons for use, and CHW feedback on the extent to which SMS capabilities facilitated and improved the quality of medical care that they could provide. Communication costs and efficiency were compared between one geographic area with SMS capabilities and two areas of similar demographics that did not have SMS or other cell phone service. In this study, CHWs most often used SMS to report medical supply shortages, followed by texts to obtain or communicate general information, and then by texts about patients with emergencies. The average cost per communication was about five times less expensive using SMS than in areas without SMS service. Communication via SMS took an average of nine minutes, whereas health workers in areas without SMS generally had to report any issues in person and this took an average of 24 hrs. Further, the CHWs using SMS claimed that they received more respect and confidence from the communities that they served and had to make fewer referrals to district hospitals since they could handle more problems on their own. It remains yet to be studied whether SMS actually reduced the incidence of supply stockouts or patient transfers [14].

An earlier retrospective study, also conducted in rural Malawi, used a free open source program called Frontline SMS to assist in the activities of 75 CHWs whose primary tasks were to manage HIV and TB patients [15]. The program uses automated responses based on key words in received text messages. Common uses were patient referral, drug dosing information, emergency support, and reporting patient mortality. Over the course of six months, it was found that the fuel savings alone (combined with those of the TB coordinator) heavily outweighed the operational costs of the FrontlineSMS network (a $2,750 net savings over six months). Similarly, the free time gained by hospital staff (2,048 hours) enabled higher resolution in data reporting (16.67 reports/week versus 25 reports/month prepilot) and expanded healthcare delivery capacity (100 additional TB patients put on treatment).

Ngabo et al. published a study on a mobile phone-based system designed and implemented in Rwanda using RapidSMS, a free and open-sourced software development framework, with the aim of monitoring pregnancy and reducing bottlenecks in communication associated with maternal and infant deaths [16]. The RapidSMS system was customized to allow interactive communication between the CHW following mother-infant pairs in their community, a national centralized database, the local health facility, and, in the case of an emergency alert, the ambulance driver. Over a 12-month period a total of 432 CHWs were trained and equipped with mobile phones by the Ministry of Health. During this time there was a registered 27% increase in facility-based delivery from 72% twelve months before to 92% at the end of the twelve-month pilot phase. The system was also found to promote prenatal visits since only registered pregnancies could be entered into the database. Further, the program facilitated the monitoring of CHW activities by remote supervisors. At the time the paper was published the project had rolled out to 18 out of the 30 districts nationwide with a total of 15,000 phones distributed to more than 7000 CHWs who were subsequently trained. Key elements that enabled the project to function were a strong commitment from the national government which provided the phones as well as covering programming fees, collaboration between the public and private sectors whereby the cost of sending text messages decreased 10-fold for all messages used in the program, and a previously well-established CHW program with good distribution of workers. Major challenges were telephone maintenance and replacement, especially in areas without electricity that were far from the nearest health centre.

A formative mixed methods study conducted by Chang et al. looks at some quantitative as well as qualitative (assessed on a Likert scale) aspects of mHealth using smartphones at a community-based HIV care organization in Uganda [17]. Interviews were conducted with 20 participants (6 CHW, 4 clinic staff, and 10 patients) and 6 focus groups. The study revealed that almost all of the participants had cell phones (not specifically smart phones) (93%), whereas almost none (4%) had access to the Internet at their homes. Most of the participants thought that using smartphones would improve the work of CHWs especially by allowing them to be monitored more effectively using GPS, photo, and video functions. Among CHWs, the main concern expressed was that the introduction of new technology might threaten their job security. Weaknesses of the study included a small sample size as well as not differentiating qualitative responses according to the role of the participant (i.e., CHW, clinic staff, and patient).

A cost analysis by Chang showed costs per patient per year in Uganda of $8.75 for a peer health worker (PHW) program versus $2.35 for an mHealth support program [18]. Both interventions were found to be reasonable for the budgetary requirements of many AIDS care programs. The effectiveness in averting virologic failure and loss to follow-up were shown for the PHW intervention with the addition of mHealth; however, a comparison of effectiveness of PHWs with and without mHealth was not performed.

Looking at the studies cited dealing with mHealth in health worker communication, it is evident that basic mobile phone use along with SMS capabilities can improve CHW efficiency while potentially reducing overall program costs. Utilization of smartphones may also be beneficial but necessitates more initial investment and has not yet shown clear advantages over basic cell phones + SMS.

## 5. eHealth

MHealth is one major component of eHealth, which refers to the utilization of information and communication technology for health more broadly, including data transmission and video telecommunication via the Internet. MHealth and Internet-based healthcare (eHealth) interventions are being used in many of the countries in SSA participating in the Millennium Villages Project. The Millennium Villages Project is using an open source eHealth platform, Global Network (MVG-Net), to track overall progress toward health-related outcomes and to help inform clinical decision making and management [19]. Key health indicators being measured include patient coverage relative to the overall population, immunizations, malnutrition screenings, and improvements in health-related outcomes. Focus is placed on the implementation of ChildCount+ (a point of care decision support SMS-based mobile phone system) and OpenMRS (a web-based, open source electronic medical record (EMR) platform). Results of the complete study are not released, but the pilot showed significant advances in the parameters described previously for those using the ChildCount+ system. Community health workers claimed to have more difficulty using the OpenMRS system indicating that more time and resources will need to be invested into training on this system before its efficacy can demonstrated. This preliminary study suggests that in SSA, the effectiveness and implementation of mHealth interventions will likely outpace eHealth. It is important to note the potential synergistic relationship between mHealth implementation and the adoption and utilization of EMR. mHealth programs being used in concert with an EMR system could potentially facilitate coordination of patient care, health worker efficiency, and data collection and analysis. However, the lack of existing infrastructure in many areas of SSA, as well as the lack of background familiarity with EMR platforms among health care workers, poses significant obstacles to the adoption of EMR systems in many areas where mHealth may be currently viable.

## 6. Health Education

A study conducted by Chib et al. in Uganda was critical of the current enthusiasm for mHealth with respect to disseminating health education materials [20]. The study focused on a Text to Change Project in the Aura district of Uganda that took place in 2009. An SMS quiz on HIV awareness was sent to 10,000 cell phone subscribers from a single provider. Questions dealt with knowledge of HIV transmission as well as questions promoting visits to the clinic for HIV testing and counselling. Of the 10,000 mobile numbers who were sent messages, 2,363 numbers responded, of which 1,954 answered the quiz questions (the rest responded to the gender and/or age questions only). Most of those who responded were men (of those who answered the gender question 421 were male versus 202 female). This was probably because men tended to be the ones with ownership of a cell phone and a higher literacy rate. This phenomenon presented a significant obstacle to the effectiveness of the program since the information was failing to reach those who are most vulnerable, that is, poor females. The study was critical of such technological interventions that fail to reach those most in need. Further, it was found that, of the question answers that were received, people tended only to answer questions that they knew the correct answer to and skip those they might not have. Thus, the amount of new information gained was likely to be limited.

## 7. eEmergency and Disaster Response

The search process used for this literature review did not reveal any formal studies of mHealth in disaster response in SSA, but there were several studies conducted in developing countries elsewhere in the world. The lessons learned in those areas could prove valuable for future applications of mHealth technology in disasters in SSA. Furthermore, in most of these studies mHealth was utilized by international humanitarian response teams, which could consider implementing these applications in future disasters in SSA.

eEmergency, or internet-based provision of emergent medical and disaster care, is expanding rapidly. Examples of the use of mobile technology in disaster response include remote triage and monitoring, telemonitoring, medical image transmission (mainly X-ray and FAST), decision support applications, field hospital IT systems, and patient and health care worker tracking. Two notable recorded instances in which mobile technologies (specifically field hospital IT systems and teleradiology) were implemented in actual disasters were the US military in Pakistan in response to the 2005 earthquake and the Israeli Defence Forces in response to the 2010 earthquake in Haiti. Both cases showed that the electronic system in use helped with management of resources, identification and tracking of patients, and continuity of care and effective discharge [21].

Another implementation study was performed on a novel electronic patient medical record and tracking system used in postearthquake Haiti in 2010 [22]. The system used iPhones and the application iChart and was implemented at the Fond Parisien (FDP) Disaster Rescue Camp for all patients registered from January to March 2010. According to the majority of the approximately 150 medical care providers who used the program, handheld EMR and tracking systems could potentially reduce workload, but the iChart program was too “cumbersome” to fulfil that need [22]. That said, the program did facilitate continuity of care and patient hand-offs by providing a centralized database (the alternatives were handwritten files or nonnetworked computers) with standardized spellings of people's names and flags for those patients that required complicated postsurgical care. Further, iChart's online database was used to generate (albeit limited) aggregate census information for real-time analysis and reporting.

It was found that important features when considering a mobile health technology to be used in disaster situations are that it be readily available (many volunteers already have iPhones), not require much training to use (iPhone apps are generally very intuitive), and have adequate data management and security (in this case the app required a user ID and pass code to log in, although the option for tiered access and permissions was not available). Another important consideration is the ability to utilize mobile devices without continuous internet connectivity, and sync when connectivity is available, preferably to a cloud-based central system [22].

Interestingly, surveys looking at user satisfaction with mHealth in disaster simulations showed that those with administrative roles tended to favour the usage of mHealth whereas providers working in the field were not as satisfied and felt that mHealth was being used to monitor them as opposed to helping them perform their duties [22]. It was also learned that mHealth technologies for disaster response would be most effective if they are utilized for everyday purposes before a disaster. Overall, more data is required to demonstrate the effectiveness of mHealth in disaster medicine, especially in less developed countries [22].

## 8. Challenges

Despite great promise, there is currently limited evidence for improved health outcomes or the costeffectiveness of mHealth in SSA. In particular, more research needs to be conducted regarding the costeffectiveness of mHealth and eHealth programs compared to other health interventions in the region. This is especially the case for underrepresented areas such as francophone Africa. Establishing feasibility still remains a priority for the field. Given the varying infrastructure throughout SSA, it is important to test the feasibility of mHealth interventions over a broad geographic distribution. Lack of standardization of mHealth applications and studies is another serious obstacle to the provision of reliable studies and data to warrant industry scaleup. The WHO has recommended moving from computer-based research, focusing on usability, to a health outcomes-based approach facilitating randomized control studies and standardized replicable study designs [5]. Another challenge is that the majority of mHealth research is being conducted in high-income countries with advanced telecommunication infrastructure, which is not yet as widely available in SSA, especially broadband internet access. This poses many problems in feasibility especially with regard to more complex mHealth programs using smart phones as well as EMRs. Some of the technical and ethical challenges facing mHealth in SSA include transmission error detection and management, ensuring patient privacy during wireless transmission [23], phone security and sharing, inconsistent or limited network availability, and specific technical issues such as movement artefact in biomonitoring [4]. Information security is of particular concern and can only be adequately addressed once there is large enough industry buy-in to facilitate standardization and policy implementation. As of yet, most existing trials are small scale with varying degrees of security measures such as firewalls and tiered password access. A study of the prospects for scaleup of mHealth specifically in South Africa by Leon et al. showed that there are barriers in the following areas: strategic leadership, learning environment, capacity for implementation, culture of information use, technology usability, interoperability, privacy and security, sustainable funding, and cost effectiveness [24]. Most prominent among these are the weaknesses in organisational capacity, culture of using health information for management, and a relatively less developed ICT environment. According to a survey by the WHO all member states in Africa, barriers to investment, and scaleup of mHealth programs in the region are primarily due to operating costs, knowledge, infrastructure, and policy (in order of magnitude) [5]. Of note, globally the primary barriers that were identified were priorities, knowledge, policy, and cost effectiveness.

## 9. Conclusions

Mobile technology has significant potential for positive impact on healthcare in SSA. Mobile infrastructure has leapfrogged land-based telecommunication infrastructure in much of the region, and mobile penetration is now high, even in low-income and remote areas. In rural areas, where population densities are lowest and access to healthcare personnel often limited, mHealth offers potential solutions for maximizing healthcare worker impact and efficiency. Areas of mHealth where the most promise has been shown to date are in medication adherence and healthcare worker communication though the evidence is not yet sufficient to warrant large-scale investment and policy change. The use of mHealth in disseminating health education anonymously has been much less successful largely due to lack of adequate penetration to the most vulnerable groups of people. Such issues of access are not so apparent in other applications of mHealth such as medication adherence and CHW communication since participants are identified beforehand and provided with the appropriate technology if they do not already have it. Internet-based applications within the scope of eHealth remain largely unfeasible in much of SSA as of yet due to lack of infrastructure as well as issues with familiarity and usability. Isolated usage of mHealth in coordination with EMR systems during disaster scenarios has had mixed results but shows great potential for coordinating patient information and improving patient care, especially if such systems are made part of day-to-day healthcare provision and thus familiar to users. While operating, costs and infrastructure remain primary obstacles to the adoption and implementation of mHealth in SSA versus other areas of the globe, the rapid expansion of market-driven telecom infrastructure in the region could overcome these hurdles in the not too distant future. Likewise, challenges resulting from lack of cultural familiarity with wireless technology are disappearing with the introduction of cell phone access in even the most remote areas of SSA. Further, the rise of 3G access and smart phones could facilitate more complex mHealth and eHealth applications as well as synchronization with EMRs even in remote areas where traditional access to the Internet has been cost prohibitive. Further progress in the field of mHealth in SSA will rely on large-scale studies demonstrating feasibility and cost effectiveness over a broad geographic distribution. Policies that, incentivize telecom companies to provide their services for mHealth programs at reduced rates would be one way of facilitating larger studies. Standardization of mHealth studies is also essential if large-scale comparative analyses are to be performed and steps should be taken by international health organizations as well as academic and industry institutions to address this challenge.
